# Exploring the potential of omega‐3 fatty acids in acne patients: A prospective intervention study

**DOI:** 10.1111/jocd.16434

**Published:** 2024-07-10

**Authors:** Anne Guertler, Katharina Neu, Diana Lill, Benjamin Clanner‐Engelshofen, Lars E. French, Markus Reinholz

**Affiliations:** ^1^ Department of Dermatology and Allergy, University Hospital LMU Munich Munich Germany; ^2^ Dr Phillip Frost Department of Dermatology and Cutaneous Surgery, Miller School of Medicine University of Miami Coral Gables USA

**Keywords:** exposome factors, Mediterranean diet, nutraceuticals, nutrition, supplementation

## Abstract

**Background:**

Omega‐3 fatty acids (ω‐3 FA), eicosapentaenoic acid (EPA), and docosahexaenoic acid (DHA), are essential nutrients known for their anti‐inflammatory properties, which involve reducing pro‐inflammatory cytokines, eicosanoids, and insulin‐like growth factor‐1. This suggests their potential to alleviate acne severity, especially when deficits are present.

**Aims:**

To elevate EPA/DHA levels in acne patients through dietary intervention and supplementation, observing subsequent clinical effects.

**Methods:**

Over 16 weeks, 60 patients without prescription medication (*n* = 23 acne comedonica [AC], *n* = 37 acne papulopustulosa [AP]) adhered to a Mediterranean diet, incorporating oral algae‐derived ω‐3 FA supplementation (600 mg DHA/300 mg EPA week 1–8, 800 mg DHA/400 mg EPA week 8–16). At four visits (V1–V4), blood EPA/DHA levels were tracked using the HS‐omega 3 index® (EPA/DHA (%) of total identified fatty acids in erythrocytes; target 8%–11%, deficit <8%, severe deficit <4%), alongside clinical assessments and standardized questionnaires.

**Results:**

At baseline, 98.3% of patients had an EPA/DHA deficit, with the mean HS‐omega 3 index® rising from 4.9% at V1 to 8.3% at V4 (*p* < 0.001). AC showed significantly higher indices than AP at V4 (*p* = 0.035). Objective improvements in both inflammatory and non‐inflammatory lesions were observed (*p* < 0.001). While self‐reported appearance worsened in four patients, overall quality of life improved (*p* < 0.001), particularly in AP. Dietary triggers were more clearly defined than beneficial foods. Intake of cow's milk and dairy products reduced (*p* < 0.001). Compliance was good; no adverse events were reported.

**Conclusion:**

Many acne patients have a ω‐3 FA deficit. The HS‐omega 3 index® can be increased by a Mediterranean diet and oral supplementation with algae‐derived ω‐3 FA. Acne severity improved significantly in patients with target ω‐3 FA levels.

## 1 INTRODUCTION

In the pursuit of skin health, and particularly in a juvenile patient cohort, such as acne vulgaris, clinicians and patients are more than ever seeking treatment approaches that go beyond the conventional options of topical and systemic prescription medications.^1^, ^2^, ^3^ As the understanding of the interplay between so‐called exposome factors and skin health deepens, there is increasing evidence pointing to the pivotal role of nutrition in shaping dermatoses.^4^ Exposome factors for acne are defined as the sum of skin care, diet, pollutants, occupational factors, climatic influences, and psychosocial mediators that influence the onset, duration, and clinical severity of the disease.^5^ Although their impact cannot be neglected, clinicians rarely educate their patients accordingly, and the potential to positively influence these factors is not being exploited.^6^, ^7^

Diet, in particular, is a variable that can be positively influenced by individuals with the appropriate knowledge. With regard to acne, ultra‐processed foods with added refined sugars/saturated fats, cow's milk, and dairy product have been implicated in the pathogenesis of acne, including sebum overproduction, altered follicular keratinization, inflammation, and follicular colonization with *Cutibacterium acnes* (*C. acnes*).^8^, ^9^, ^10^, ^11^ The implementation of dietary interventions with nutrients that may alleviate acne severity as an adjunctive, feasible treatment option is promising, but scientific evidence remains scarce. In this context, Omega‐3 (ω‐3) fatty acids (FA), including alpha‐linolenic acid (ALA), eicosapentaenoic acid (EPA), and docosahexaenoic acid (DHA), have been proposed as promising agents due to their diverse biological functions.^12^, ^13^ While ALA is an essential FA, meaning that it must be obtained from the diet, EPA and DHA can be biosynthesized from ALA, albeit in very small amounts. Food and supplements are therefore necessary sources for all ω‐3 FA. Recent studies suggest that daily ω‐3 FA requirements may vary between age, gender, and life circumstances such as pregnancy and breastfeeding; however, target values are rarely assessed.^14^ According to the European Food Safety Authority (EFSA), the daily intake through oral supplementation should not exceed 5 g, while the Food and Drug Administration (FDA) advises a daily maximum of 3 g daily.^15^ Balancing the intake of these fatty acids has significant clinical implications. Particularly in Western countries, where the common diet is characterized by an excess of ω‐6 FA, compared to ω‐3 FA. Whereas humans used to consume ω‐3 FA and ω‐6 FA in a ratio of approximately 1:5, modern diets have a ratio as high as of 1:20 or more.^16^ While both are essential for our overall health, ω‐6 FA promote inflammatory pathways when consumed in excess. The main type of ω‐6 FA is linoleic acid (LA), which is commonly found in vegetable oils and therefore in fried foods, can be converted to arachidonic acid (AA), a precursor of inflammatory eicosanoids.^17^ A balanced ratio may not only reduce inflammation in general, but specifically inflammatory dermatoses, including acne.^18^, ^19^

Several mechanisms have been proposed to elucidate the potential impacts of ω‐3 FA on acne. These include modulation of sebum production, reduction of inflammatory cytokines, inhibition of *C. acnes* growth, improvement of skin barrier function, and antioxidant properties.^12^, ^13^, ^20^, ^21^ Insulin‐like growth factor (IGF)‐1 signaling stands out as a pivotal acne pathway, inducing an array of pro‐inflammatory cytokines and matrix metalloproteinase (MMP) in human sebocytes. This pathway also activates the nutrient‐sensitive kinase mammalian target of rapamycin complex (mTORC1), which is intertwined with anabolic pathways, including sebaceous lipogenesis and follicular hyperkeratosis, all of which play key roles in the pathogenesis of acne vulgaris.^10^, ^22^ ω‐3 FA have shown to decrease serum levels of IGF‐1 while increasing IGF binding protein‐3 (IGFBP‐3). This prevents IGF‐1 from binding to its receptors on sebocytes and acroinfundibular keratinocytes.^10^, ^23^ Moreover, ω‐3 FA have exhibited the capacity to inhibit toll‐like receptor (TLR)‐signaling pathways, with both DHA and EPA leading to a decreased inflammatory response in keratinocytes and macrophages.^24^ Consequently, this results in a reduced activation of nuclear factor‐kappaB (NF‐ κB) and mitogen‐activated protein kinase (MAPK) pathways, subsequently curbing the production of interleukins (IL)‐1, IL‐6, IL‐8, tumor‐necrosis factor (TNF)‐alpha, and MMP production by ω‐3 FA.^25^, ^26^ Furthermore, ω‐3 FA have inhibitory effects on the NLRP3 inflammasome, a cytoplasmic multiprotein complex that can be initiated in response to various signals, including those of *C. acnes*.^27^, ^28^ This inhibition ultimately leads to a decreased caspase‐1 activation with reduced IL‐1beta‐driven Th17 activation, which contributes to acne's inflammation and dyskeratinization.^29^

Although preliminary studies suggest a potential benefit of ω‐3 FA in acne, the data remain inconclusive due to limited study designs and, most importantly, a failure to address existing deficits.^12^, ^13^, ^30^, ^31^, ^32^, ^33^

The aims of this prospective 16‐week study were to assess EPA/DHA levels in acne patients using the HS‐omega‐3 Index® in erythrocytes (%), to raise levels to the target range (HS‐omega‐3 Index® 8%–11%) using dietary recommendations based on current evidence (plant‐focused Mediterranean diet with limited intake of milk/dairy/ultra‐processed ingredients) and oral supplementation with algae‐derived EPA/DHA, and to assess the effect on clinical severity. The findings may reshape future treatment protocols and enable both healthcare providers and patients alike to contribute to clinical outcomes.

## 2 MATERIALS AND METHODS

### 2.1 Study design

This single‐center intervention study was conducted at the Department of Dermatology and Allergy, Ludwig‐Maximilians‐Universität, Munich, Germany. Patients with mild (acne comedonica = AC) to moderate (acne papulopustulosa = AP) acne vulgaris were enrolled for a period of 16 weeks, with four study visits ([V]: V1 = baseline, V2 = week 6, V3 = week 12, and V4 = week 16). Inclusion criteria were patients aged 12 years and older, not using any prescription acne medication 4 weeks prior to study inclusion and throughout the study. Daily skin care use consisting of a wash lotion and moisturizer was allowed. Participants, parents, or respective legal guardians of minors provided written informed consent prior to study inclusion. Exclusion criteria were a history of eating disorders and psychological comorbidities, pregnancy, and breastfeeding, as well as acne patients with severe clinical presentation (acne conglobata) or patients who wanted or needed prescription medication (either topical or systemic). Ethical approval was granted by the Ethics Committee of the Medical Faculty of the Ludwig Maximilian University (LMU), Munich Germany (ref. no. 20‐0855).

### 2.2 Intervention

#### 2.2.1 Dietary recommendations

At each study visit, participants received individual counseling, including education about exposome factors and their potential impact on acne severity. Particular emphasis was placed on diet. Patients were encouraged to follow a plant‐focused, varied Mediterranean diet, consisting mainly of unprocessed, seasonal, nutrient‐dense ingredients, and to prepare home‐cooked meals. They were advised to reduce their intake of ultra‐processed foods, milk/dairy products, and meat.^8^, ^20^, ^34^ Adherence to the dietary recommendations was recorded using standardized food frequency questionnaires (see below).

#### 2.2.2 Omega‐3 fatty acids

Oral supplementation with DHA and EPA from *Schizochytrium* sp. algae was recommended.^35^ (Omega‐3 pflanzlich + D vegan Doppelherz® Queisser Pharma GmbH & Co. KG, Flensburg, Germany; one capsule containing 200 mg DHA/100 mg EPA). From V1 to V2, each patient was advised to take three capsules daily with the main meal of the day (600 mg DHA/300 mg EPA daily), and from V2 to V4 four capsules daily (800 mg DHA/400 mg EPA daily).

### 2.3 Assessments and outcomes V1–V4

#### 2.3.1 Laboratory analysis

Blood samples were taken according to a standardized protocol. A detailed laboratory analysis was performed including EPA/DHA assessment using the HS‐omega‐3 Index® (Omegametrix® GmbH, Martinsried, Germany). For the HS‐omega‐3 Index®, fatty acid methyl esters were prepared from erythrocytes by acid transesterification and analyzed by gas chromatography using a GC2010 Gas Chromatograph (Shimadzu, Duisburg, Germany), equipped with a SP2560, 100‐m column (Supelco, Bellefonte, Pennsylvania) using hydrogen as carrier gas. Fatty acids were identified by comparison with a standard mixture of fatty acids characteristic of erythrocytes. HS‐omega‐3 Index® results were expressed as EPA plus DHA as a percentage of total identified fatty acids after response factor correction. The coefficient of variation for EPA plus DHA was 5%. Analyses were quality‐controlled according to DIN ISO 15189. The HS‐omega‐3 Index® accurately reflected the ω‐3‐FA status of a human individual.^36^ The target range of the HS‐omega‐3 Index® was defined as 8%–11%, with values between 4% and 8% defined as a deficit, and values <4% defined as a severe deficit.^37^

#### 2.3.2 Clinical assessment

The predilection sites of acne (face, chest, and back) were examined and the clinical severity was classified by an independent dermatologist at each visit using the Global Investigator Assessment (IGA). Based on the count of noninflammatory (comedones) and inflammatory lesions (papules and pustules), acne was divided into its main subtypes: acne comedonica, acne papulopustulosa, and acne conglobata.^38^ Severity of AC and AP was further assessed using a 3‐point scale (mild, intermediate, and severe). The appearance of the skin was also self‐assessed by the patients in terms of overall severity and extent of noninflammatory and inflammatory lesions using a 3‐point scale (mild, intermediate, and severe, respectively). Professional photographers documented the clinical presentation at V1 and V4 using digital photography (Nikon D5, AF‐S Nikkor 60 mm lens).

#### 2.3.3 Sebum levels

The Sebumeter® SM 815 (Courage + Khazaka, Cologne, Germany) was used to quantitatively measure patients' facial sebum levels. The handheld device, which exposes a 64 mm^2^ strip of tape, was placed perpendicularly and without any pressure on three predefined areas of the patients' skin (forehead and both cheeks). The degree of tape transparency correlated with skin sebum levels (μg/cm^2^), determined by photometry.^39^ Measurements were performed under standardized conditions in an air‐conditioned room.

#### 2.3.4 Questionnaires

At V1, a detailed investigator‐developed questionnaire was used to obtain information on patient demographics and lifestyle aspects. Patients were also asked to rank various foods according to whether they aggravated or helped the severity of their acne.

At V1–V4, patients' dietary intake over the previous 4 weeks was assessed using a standardized food frequency survey (FFS).^40^, ^41^ Patient records were reviewed by an independent clinical nutritionist to monitor adherence to dietary recommendations.

The Dermatology Life Quality Index (DLQI) was used at V1–V4 as a standardized tool to assess the impact of acne on patients' quality of life including psychological disability at work, social and sexual relationships, depression, and anxiety. The higher the score, the more the patient's quality of life was affected, with scores ranging from 0 to 30 (0–1 no effect, 2–5 small effect, 6–10 moderate effect, 11–20 very large effect, and 21–30 extremely large effect).^42^

### 2.4 Primary objective

The primary objective was to measure the HS‐omega‐3 Index® in acne patients and to raise the mean levels of the cohort to the target range through dietary recommendations including oral supplementation with EPA/DHA over the course of 16 weeks. A mean HS‐omega‐3 Index® between 8% and 11% at V4 was defined as a successful intervention.^43^

### 2.5 Secondary objectives

Secondary objectives were defined as follows:

- To evaluate the clinical severity of acne
- To assess acne patients' facial sebum values
- To investigate acne patients' dietary habits
- To rate participants' quality of life

### 2.6 Statistical evaluation

A professional statistician performed all data analyses using SPSS software version 26 (IBM, Armonk, New York). A *p*‐value of 0.05 was set as the level of significance for all statistical tests.

Data from study dropouts were included in accordance with ethical approval. Continuous variables were summarized using descriptive statistics, including number of subjects (*n*), mean, standard deviation (SD), median, minimum, maximum, and 95% confidence interval. For categorical variables, summaries included counts of subjects and percentages in corresponding categories. Multivariate logistic regression analysis was used to identify associations between variables and acne severity. Statistical tests such as Mann–Whitney *U* test, Wilcoxon test, chi‐squared test, Fisher's exact test, McNemar test, and binomial tests were used where appropriate.

## 3 RESULTS

### 3.1 Demographic data

The study cohort comprised 60 patients (*n* = 23 AC, *n* = 37 AP) with a mean age of 26.1 ± 4.7 years and a mean acne persistence for 8.9 ± 5.5 years. More than half of the patients reported low satisfaction with previous treatments (*n* = 38, 63.3%), mostly because of insufficient clinical improvement (*n* = 32, 84.2%) and side effects (*n* = 30, 78.9%), while 36.7% were satisfied with previous therapies. Demographics are shown in Table 1.

**TABLE 1:** Patients' demographics.

	Total cohort (*n* = 60)
Gender (*n* (%))	
Male	2 (3.3)
Female	58 (96.7)
Mean age (years (SD))	26.1 (4.7)
Mean BMI (kg/(m)^2^)	21.8 (2.9)
Acne subtype (*n* (%))	
Acne comedonica	23 (38.3)
Acne papulopustulosa	37 (61.7)
Mean acne persistence (years (SD))	8.9 (5.5)
Affected anatomic sites (*n* (%))	
Face	60 (100)
Chest	29 (48.3)
Back	35 (58.3)
Skin type (*n* (%))	
Normal	3 (5)
Dry	6 (10)
Mixed	43 (71.7)
Oily	8 (13.3)
Daily skin care use (*n* (%))	
Wash lotion	42 (70.0)
Moisturizer	44 (73.3)
Medical history (*n* (%))	
Thyroid disease	7 (11.7)
Other skin disease	7 (11.7)
Smoking	7 (11.7)
IBS	4 (6.7)
Diabetes	1 (1.7)
PCOS	1 (1.7)
Highest education (*n* (%))	
University degree	24 (40.0)
High school degree	14 (23.3)
Other	21 (36.7)

Abbreviations: BMI, body‐mass index; IBS, irritated bowel syndrome; kg, kilogram; m, meter; *n*, number; SD, standard deviation.

### 3.2 HS‐Omega‐3 Index®

At baseline, 98.3% of the cohort showed a deficit in EPA/DHA according to the HS‐omega‐3 Index® (*n* = 40 deficit, *n* = 18 severe deficit). Over time, the mean HS‐omega‐3 Index® increased significantly from 4.9% at V1 to 8.3% at V4 (*p* < 0.001). At V4, patients with AC had a significantly higher HS‐omega‐3 Index® compared to AP (*p* = 0.035). (Table 2).

**TABLE 2:** Overview of patients' HS‐omega‐3 Index® throughout the study.

	V1 (*n* = 59)	V2 (*n* = 55)	V3 (*n* = 53)	V4 (*n* = 53)	*p* Value*
Severe deficit (HS‐omega‐3 Index® < 4%), *n* (%)	18 (30.5)	1 (1.8)	1 (1.9)	1 (1.9)	<0.001
Deficit (HS‐omega‐3 Index® 4–8%), *n* (%)	40 (67.8)	44 (80.0)	22 (41.5)	18 (33.9)	<0.0
No deficit (HS‐omega‐3 Index® >8%), *n* (%)	1 (1.7)	10 (18.2)	30 (56.6)	33 (62.3)	<0.001
Mean HS‐omega‐3 Index® (%)	4.9	6.8	8.0	8.3	<0.001

Abbreviations: *n*, number; SD, standard deviation; V, visit.

* Statistical significance V1–V4.

### 3.3 Clinical severity

Acne severity improved significantly over the course of the study, with a reduction in the absolute number of noninflammatory and inflammatory lesions between V1 and V4 according to dermatologic objective assessment (*p* < 0.001). At V4, more patients presented with AC than AP (Figure 1, Table 3).

**FIGURE 1:**
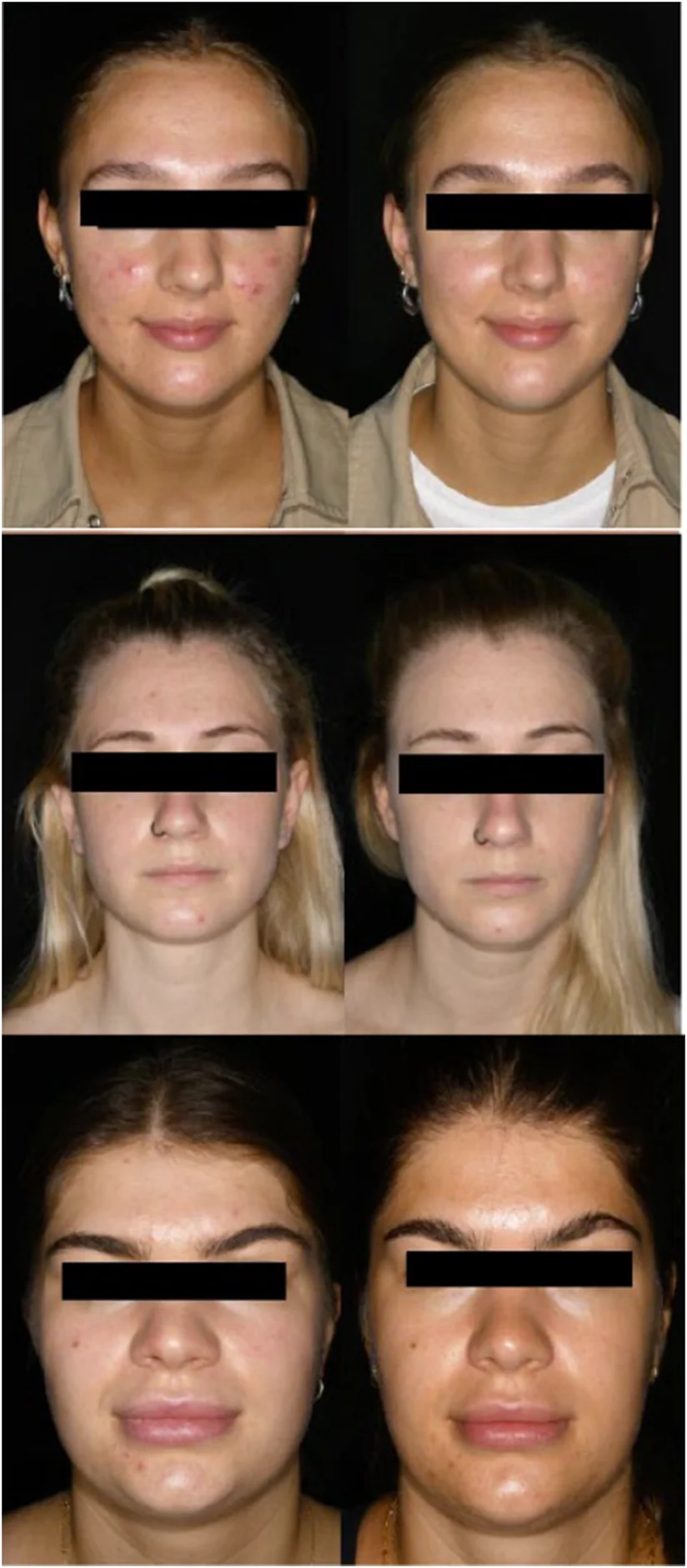
Comparison of clinical severity of three patients between V1 (left pictures) and V4 (right pictures).

**TABLE 3:** Clinical acne assessment.

	V1 (*n* = 60)	V2 (*n* = 55)	V3 (*n* = 53)	V4 (*n* = 53)	*p* Value*
Acne subtype (*n* (%))					
Acne comedonica	23 (38.3)	39 (70.9)	41 (77.4)	42 (79.2)	<0.001
Acne papulopustulosa	37 (61.7)	16 (29.1)	12 (22.6)	11 (20.8)	<0.001
Clinical severity (*n* (%))					
Mild	29 (46.7)	50 (90.9)	49 (92.5)	45 (84.9)	<0.001
Intermediate	32 (53.3)	5 (9.1)	4 (7.5)	8 (15.1)	<0.001
Affected anatomic sites (*n* (%))					
Face	60 (100)	55 (100)	53 (100)	52 (98.1)	0.316
Chest	29 (48.3)	6 (10.9)	8 (15.1)	6 (11.3)	<0.001
Back	35 (58.3)	16 (29.1)	12 (22.6)	7 (13.2)	<0.001
Noninflammatory lesions (*n* (%))					
None				2 (3.8)	
≤10	8 (13.3)	35 (63.6)	42 (79.2)	42 (79.2)	<0.001
10–25	27 (45)	18 (32.7)	10 (18.9)	8 (15.1)	<0.001
26–50	20 (33.3)	2 (3.6)	1 (1.9)	1 (1.9)	<0.001
≥ 50	5 (8.3)				
Inflammatory lesions (*n* (%))					
None		10 (18.2)	11 (20.8)	13 (24.5)	
≤10	23 (38.3)	38 (69.1)	39 (73.6)	33 (62.3)	<0.001
10–20	28 (46.7)	5 (9.1)	3 (5.7)	7 (13.2)	<0.001
21–30	8 (13.3)	2 (3.6)			
≥ 30	1 (1.7)				

Abbreviations: *n*, number; V, visit.

* Statistical significance V1–V4.

Comparing V1 and V4, the self‐reported acne severity improved according to 42 patients (79.2%), remained unchanged in seven (13.2%), and worsened in four (7.5%). Subjective clinical improvement was reported after an average of 40 days, in the number of comedones (*n* = 23, 37.1%), pustules (*n* = 39, 62.9%), and papules (*n* = 25, 40.3%) comparing V1 and V4 (*p* < 0.001, respectively).

### 3.4 Sebum

Patients' facial sebum levels were positively correlated with the number of comedones at V1 (*p* = 0.017, *r* = 0.306), while no significant correlation was found for inflammatory skin lesions. Absolute facial sebum levels decreased throughout the study from 179.35 μg/cm^2^ at V1 to 153.1 μg/cm^2^ at V4, although not significantly (*p* = 0.165).

### 3.5 Diet

Assessment of foods at V1 with self‐reported positive and negative effects on acne severity revealed that vegetables (*n* = 14/60, 23%), fruit (*n* = 12/60, 20%), nuts (*n* = 7, 11.7%), and whole grains (*n* = 7, 11.7%) were considered the most beneficial while refined sugar (*n* = 32/60 53.3%), alcohol (*n* = 32/60 53.3%), chocolate (*n* = 31/60 51.7%), cow's milk (*n* = 28/60 46.7%), and saturated fats (*n* = 28/60 46.7%) were perceived as the most aggravating factors. Overall, dietary acne triggers were more clearly defined than beneficial foods. (Figure 2).

**FIGURE 2:**
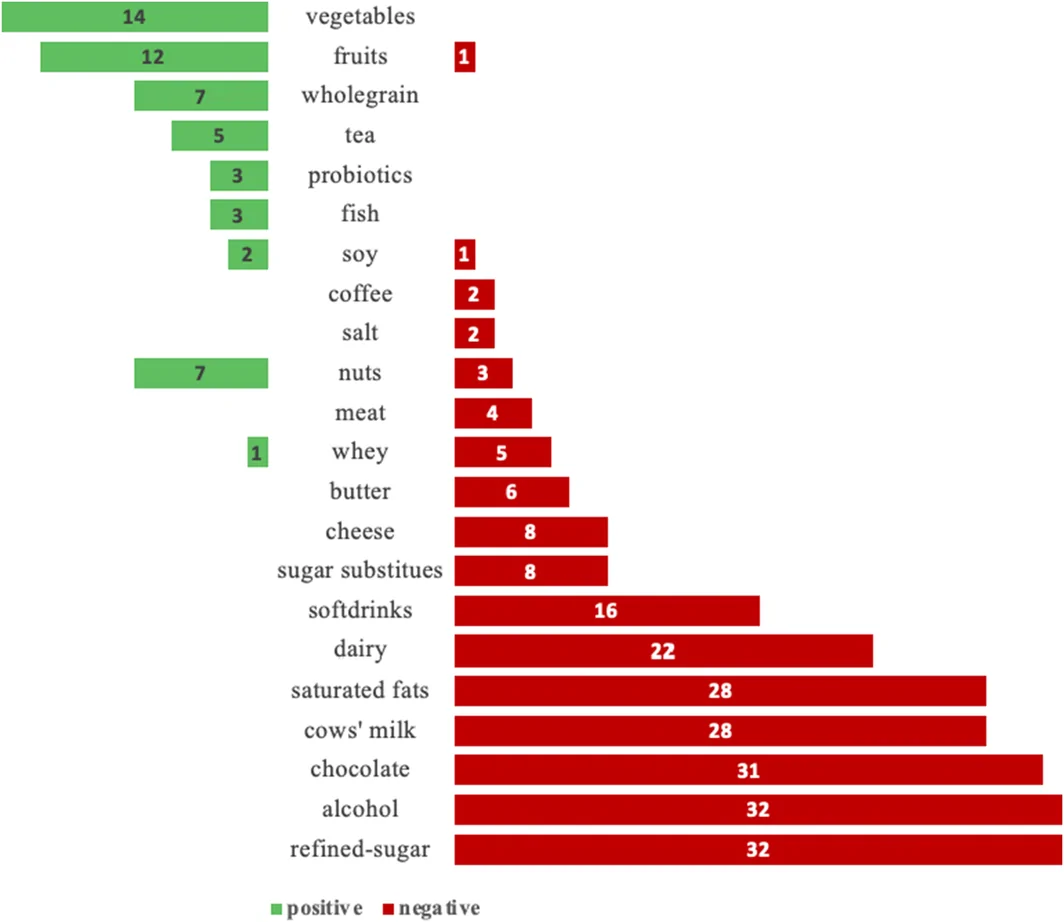
Foods with self‐reported positive (green) and negative (red) effect on acne severity. (Descriptive results are expressed as absolute number of patients identifying each item at V1.)

Evaluation of patients' FFS revealed that AP consumed significantly more cow's milk (*p* = 0.017), cornflakes (*p* = 0.017), fries (*p* = 0.041), and potato chips (*p* = 0.04) at V1 compared to the AC group, while no significant difference was found for other foods. Cow's milk and dairy products were less frequently consumed across all patients comparing V4 to V1 according to reports of the FFS (*p* < 0.001).

### 3.6 Quality of life

Mean DLQI scores decreased from 4.9 at V1 to 2.4 at V4 (*p* < 0.001). Improvement was more pronounced in the AP group compared to AC (*p* = 0.006). The more severe the clinical presentation as assessed by dermatologic evaluation, the higher the DLQI scores at V1 (*p* = 0.008) and V4 (*p* = 0.043).

### 3.7 Compliance and safety

Overall, adherence to dietary recommendations was good, except for one patient who reported not taking DHA/EPA supplements regularly. Eight dropouts were reported during this study (*n* = 3 for personal reasons, *n* = 2 for subjective worsening of acne, *n* = 2 for pregnancy, and *n* = 1 loss to follow‐up). No other adverse events were observed.

## 4 DISCUSSION

While prescription medications are mandatory for the treatment of acne vulgaris, especially in severe clinical presentations, to avoid unnecessary prolongation of suffering and long‐term sequelae of scarring,^44^ the potential of lifestyle interventions should not be neglected. In particular, dietary recommendations, as they are not contradictory to conventional medicine, but rather a valuable adjunct to any modern treatment plan.^7^, ^8^, ^20^, ^45^, ^46^ This prospective study investigated the benefits of targeting ω‐3 FA levels in patients with mild to moderate acne vulgaris without prescription medication. Many of the acne patients studied had a severe EPA/DHA deficit at baseline, with an average HS‐omega 3 index® of 4.9%, which was increased to a target range of 8.3% over the course of 16 weeks. Throughout the study, patients were encouraged to follow a varied, plant‐focused Mediterranean diet and to take oral supplements of algae‐derived DHA/EPA.

A significant improvement in the number of noninflammatory and inflammatory acne lesions was seen between V1 and V4 (*p* < 0.001). At the end of the intervention, a significantly higher HS‐omega 3 index® was observed in patients with AC compared to AP. This finding may be explained by the proposed anti‐inflammatory properties of ω‐3 FA,^47^, ^48^, ^49^ corresponding to clinically reduced inflammation in patients with higher levels. There was no correlation between facial sebum levels and the HS‐omega‐3 Index®, but there was a correlation with the number of comedones, highlighting the known pathophysiological link between seborrhea as the driving force for these initial acne lesions.

While the mean HS‐omega‐3 Index® of the entire cohort reached the target value when comparing V1 and V4, some participants did not respond adequately within 16 weeks. Noncompliance, as reflected in patients' FFS, is a possible explanation. In addition, there may be differences in the individual response of patients to ω‐3 FA supplementation.^32^ Although patients' were advised to take EPA/DHA supplements in addition to an adequate fat intake, the bioavailability of ω‐3 FA supplements may vary between individuals.^32^ Future studies may need to adjust the dosage according to the individual HS‐omega‐3 Index® in order to reach the target range in all patients. It will also be of interest to determine whether blood levels at the upper end of the target range, around 11%, confer any clinical benefit. This could be achieved either by prolonged supplementation or by higher doses of DHA/EPA, especially as there have been no adverse events associated with the current supplementation.

Interestingly, patients reported the most significant clinical change after an average of 40 days. By this time (between V2 and V3), the dosage of EPA/DHA supplementation had been increased and the HS‐omega‐3 Index® had increased overall, with only one patient still having with a severe deficit. This again supports the hypothesis of clinical benefit from supplementation of deficits. Although most patients benefited, three patients reported a subjective worsening of their acne during the study, and two withdrew from the study due to worsening. It is therefore important to tailor treatment plans individually, especially when there is an inadequate response to treatment, and to always offer patients prescription medication when needed.

The assessment of EPA/DHA blood levels in this study provides unprecedented data from a German acne cohort. While previous data show a general tendency toward a ω‐3‐FA deficit in the European population, exemplarily with a HS‐omega 3 index® below 8% in 76.15% of a cohort of 23.615 European volunteers,^32^ and a mean value of 5.49% observed in a German nationwide cohort of middle‐aged women,^50^ our study suggests an exacerbation in acne patients. To date, only a few prospective, intervention human studies have been conducted to investigate the clinical effects of ω‐3 FA on acne, and remarkably, none of these have investigated patient blood levels.^13^ Rubin et al.^30^ conducted a study in 2008 with five acne patients. After 8 weeks of EPA supplementation, combined with a green tea antioxidant, the number of inflammatory facial lesions decreased, and the patients' self‐rated mental status improved. In 2012, Khayef et al.^31^ investigated the effects of DHA and docosapentaenoic acid (DPA) in 13 men. In addition to the number of facial lesions, the authors assessed patients' skin redness and evaluated a 3‐day dietary diary. No significant differences were found when comparing the number of lesions at baseline and 12 weeks after the intervention. Jung et al. conducted the highest quality intervention study to date, including histopathologic analysis of facial lesions after 12 weeks of oral ω‐3‐FA/ω‐6‐FA supplementation, which impressively demonstrated a reduction in inflammatory markers.^12^ With these limited and sometimes conflicting data in mind, this study adds new data on EPA/DHA deficits in acne patients and shows what can be achieved by dietary intervention with oral supplementation.

This study also included a subjective assessment of dietary acne triggers and beneficial foods. Defined dietary acne triggers were consistent with the current literature.^51^, ^52^, ^53^ Vegetables, fruit, whole grains, and nuts were perceived as the most beneficial. A possible explanation for the attributed positive effect of nuts on acne severity may be due to their high levels of ω‐3 FA.^21^ All these foods also comprised the recommended plant‐focused Mediterranean diet. Overall, the fact that triggers were more clearly defined than beneficial foods underlines the importance of this study, which aimed to identify nutrients that might alleviate acne severity.

As this study did not include any prescription drugs, clinical changes need to be assessed realistically. (Figure 1) However, even a small clinical improvement led to a significant improvement in the quality of life of affected patients as seen by the DLQI results and underlines the importance of incorporating lifestyle interventions. In our experience, investing time in individualized (nutritional) counseling and patient education, especially at the first visit, whether as a stand‐alone treatment or as a basis for prescription medication, not only increases patients' ability to contribute to their health, but also improves overall treatment satisfaction and outcome.

As with previous studies investigating the effects of diet on clinical parameters in acne patients, the challenge of this study remains to establish direct causal relationships as to whether increased EPA/DHA levels, changed dietary habits, or other cofactors led to the improved clinical severity of acne and improved quality of life in patients. This study therefore has its strengths and limitations. Unfortunately, a randomized, placebo‐controlled design, although initially intended, was not approved by the ethics committee at the time of the study. In addition, relying on FFS to assess patients' adherence to dietary recommendations imposes potential recall bias. Ideally, meals and supplements should be provided to each participant throughout the study period in a controlled environment throughout the study, but this is not feasible over 16 weeks. Study dropouts due to worsening clinical appearance should be addressed in future studies by reanalyzing patients' EPA/DHA levels to rule out insufficient increases and to allow individual adjustment of EPA/DHA supplement dosage. Another limitation is the predominance of female participants. As the recruiting clinic primarily serves patients with severe clinical presentations requiring prescription medication, it is worth noting that male patients, especially at a younger age, often have a more severe clinical presentation, which may explain why most male patients were not eligible to participate without prescription medication. In addition, in our experience, women tend to be more willing to participate in clinical trials and are generally more motivated to engage in lifestyle interventions, including dietary changes. While we recognize this gender imbalance, we hypothesize that it may not have significantly impacted the current results. This is supported by the observation that both male patients had an ω‐3 FA deficit at baseline, and existing data suggest that EPA/DHA deficits may occur in humans regardless of gender.^32^ Future studies should build on the foundation laid by our current findings in a randomized, placebo‐controlled design to improve dietary recommendations for acne patients.

## 5 CONCLUSION

Many patients with acne have an EPA/DHA deficit. Lifestyle interventions, including a plant‐focused Mediterranean diet and EPA/DHA supplementation for 16 weeks, led to a significant increase in ω‐3 FA levels, as assessed by the HS‐omega‐3 Index®, in patients with mild to moderate acne. Improvements in clinical appearance and quality of life were observed. While this study evaluated the approach as a stand‐alone treatment, future studies should investigate ω‐3 FA as an adjunct to any acne treatment plan.

## AUTHOR CONTRIBUTIONS

Conceptualization: AG, DL, and MR. Methodology: AG and KN. Validation: MR and LEF. Formal analysis: AG, MR, and KN. Investigation: KN and AG. Data curation: KN, AG, and MR. Writing—original draft preparation: AG. Writing—review & editing: AG, KN, DL, BCE, LEF, and MR. Visualization: AG, KN, DL, and BCE. Supervision: MR and LEF.

## FUNDING INFORMATION

No funding was received. Omegametrix® GmbH performed the analysis of the HS‐omega‐3 Index® free of charge. Queisser Pharma GmbH & Co. KG offered omega‐3 supplements free of charge. Neither Omegametrix® GmbH, nor Queisser Pharma GmbH & Co. KG had any role in the study design, evaluation, or interpretation of data.

## CONFLICT OF INTEREST STATEMENT

All authors declare that they have no competing interests.

## CONSENT FOR PUBLICATION

For all personal information used in this article, for example, personal images, a consent for publication has been obtained.

## Data Availability

The datasets used and/or analyzed during this study are available from the corresponding author on reasonable request.
